# Integrative Analysis of Transcription Factor Combinatorial Interactions Using a Bayesian Tensor Factorization Approach

**DOI:** 10.3389/fgene.2017.00140

**Published:** 2017-09-28

**Authors:** Yusen Ye, Lin Gao, Shihua Zhang

**Affiliations:** ^1^School of Computer Science and Technology, Xidian University, Xi'an, China; ^2^NCMIS, CEMS, RCSDS, Academy of Mathematics and Systems Science, Chinese Academy of Sciences, Beijing, China; ^3^School of Mathematical Sciences, University of Chinese Academy of Sciences, Beijing, China

**Keywords:** transcription regulation, TF regulatory networks, tensor factorization, integrative analysis of omics data, biological networks

## Abstract

Transcription factors play a key role in transcriptional regulation of genes and determination of cellular identity through combinatorial interactions. However, current studies about combinatorial regulation is deficient due to lack of experimental data in the same cellular environment and extensive existence of data noise. Here, we adopt a Bayesian CANDECOMP/PARAFAC (CP) factorization approach (BCPF) to integrate multiple datasets in a network paradigm for determining precise TF interaction landscapes. In our first application, we apply BCPF to integrate three networks built based on diverse datasets of multiple cell lines from ENCODE respectively to predict a global and precise TF interaction network. This network gives 38 novel TF interactions with distinct biological functions. In our second application, we apply BCPF to seven types of cell type TF regulatory networks and predict seven cell lineage TF interaction networks, respectively. By further exploring the dynamics and modularity of them, we find cell lineage-specific hub TFs participate in cell type or lineage-specific regulation by interacting with non-specific TFs. Furthermore, we illustrate the biological function of hub TFs by taking those of cancer lineage and blood lineage as examples. Taken together, our integrative analysis can reveal more precise and extensive description about human TF combinatorial interactions.

## Introduction

TRANSCRIPTION factors (TFs) are believed in playing a key role in transcriptional regulation of genes and determination of cellular identity and functions (Yu et al., 2006; Vaquerizas et al., 2009). Individual TFs participate in controlling the expression of a number of target genes by interacting with other TFs directly or indirectly. That is, multiple TFs act in concert to regulate living activities (Yu et al., 2006; Kazemian et al., 2013).

In the past decade, several methods have been proposed to find cooperative TFs. For example, Walhout and his group depicted a TF interaction network by protein-DNA and protein-protein interaction mapping (Walhout, 2006). GuhaThakurta and Stormo developed a method Co-Bind to identify DNA target sites for co-binding TFs using a Gibbs sampling strategy (GuhaThakurta and Stormo, 2001). Yu et al. predicted TF interaction pairs by the analysis of the promoter regions of specific expression genes across all known sequence-specific TFs (Yu et al., 2006). Kazemian et al. revealed a lot of TFs clusters, each of which consists of one and more non-redundant TF pairs at similar genomic loci (Kazemian et al., 2013). However, these methods may need lots of computing time and cause many non-functional matches.

The success of high-throughput sequencing technology, especially the rapid accumulation of ChIP-seq makes it possible to identify genome-wide TF-binding signals and find co-occupancy interactions with fairly high accuracy and low complexity. For example, Chikina and Troyanskaya presented a method for testing the similarity of peak distributions by comparing each single peak region of a query TF with those of a reference TF. Carstensen et al. proposed a method of distance-based measures to detect TF interactions (Chikina and Troyanskaya, 2012; Chakrabarti et al., 2015). Teng et al. developed a probabilistic program to identify frequent combinatorial occupancy patterns by taking into account uncertainty in TF and chromatin modification datasets (Teng et al., 2014). Moreover, Griffon et al. produced co-occupancy networks to identify cell-specific combinations among TFs (Griffon et al., 2015). However, due to the difficulty of detecting the activities of multiple TFs in the same cellular environment, the complexity of ChIP-seq processing and the difference of quality assessment criteria, there must exist many missing entries and lots of noise. Previous methods are difficult to offer an accurate and global landscape of TF combinatorial interactions.

Recently, several studies stated that the expression profiles of TF interacting genes tend to be highly correlated compared to others (Djekidel et al., 2015). Co-expression networks have been used to detect chromatin maintainer networks and predict TF interactions (Djekidel et al., 2015). Also, co-methylation of TF gene promoter regions have a similar effect on TF interactions. Moreover, Neph et al. reported 41 human cell-specific regulatory networks using DNase I footprinting technique, which suffers from distinct false positive regulations and a lot of noise due to serious non-functional matching for motif scanning (Neph et al., 2012). These studies provide valuable opportunities for us to integrate multiple interaction information and analyze cell lineage-specific TF interactions (Zhang et al., 2014). The integrative analysis of multiple datasets with distinct incompleteness and noise poses new challenges to predict whole and accurate combinatorial TF interactions.

**Figure 1:**
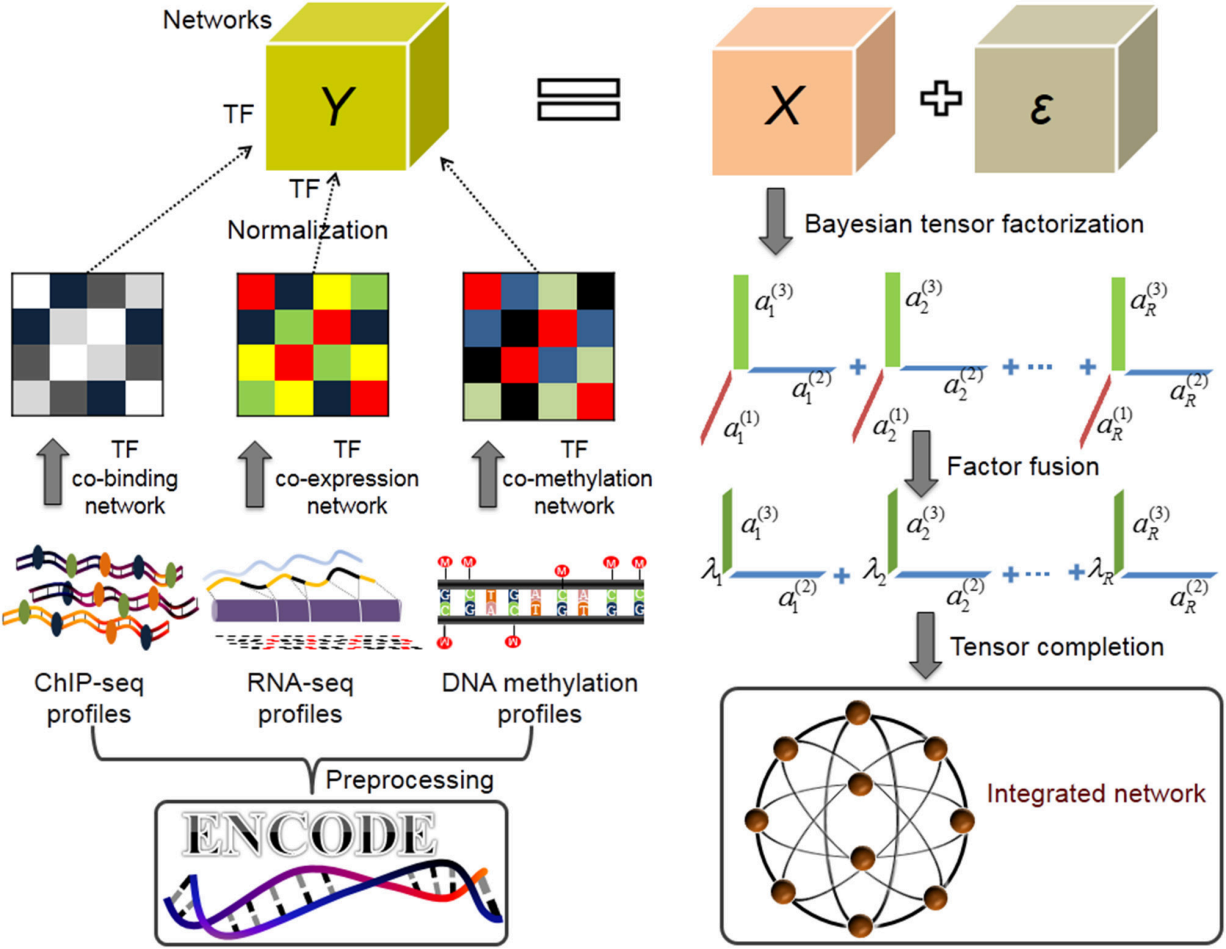
Overview of inferring TF combinatorial interactions (Application I). After data preprocessing, we first merge overlapping binding sites of the same TF in various conditions based on 155 TFs ChIP-seq datasets from ENCODE project and get the discrete, non-redundant genome binding region for each TF. We then construct a co-binding network by calculating the percentage of significant overlaps between the non-redundant binding sites of each pair of TFs, and build co-expression and co-methylation networks using PCC, respectively. We normalize the three networks respectively and stack them into a tensor. We next infer the multilinear factors over the tensor using BCPF. Finally, we fuse factors of network dimensions into a weight vector λ_*i*_, ∀*i* ∈ [1, *R*] for every rank-one matrix of TF interactions and obtain a weighted TF interaction network.

To this end, we adopt and extend a powerful Bayesian CANDECOMP/PARAFAC (CP) factorization method (BCPF) (Zhao et al., 2015) to integrate multiple TF interaction datasets in a network paradigm. BCPF effectively decomposes incomplete and noisy tensor into multiple principal multilinear components with low-rank constraint, each of which consists of three vectors and indicates the relative contribution scores of each elements in three dimensions. Generally, this problem can be consider as a tensor completion problem, which is an extension of matrix completion for high-order data. We can obtain a precise prediction for any missing entries of the tensor indicated by the corresponding factor contribution scores of the entry, and further gain the global network precisely (Figure 1).

In our first application, we apply BCPF to integrate three networks (TF co-binding, co-expression, co-methylation networks) built based on ChIP-seq, RNA-seq, and DNA methylation datasets of multiple cell lines from ENCODE respectively to predict a global and precise TF interaction network. This network gives 38 novel TF interactions with distinct biological functions which offers new clues for further study. In our second application, we apply BCPF to seven types of cell type TF regulatory networks [including seven blood (BL) networks, two cancer (CA) networks, four endothelia (EN) networks, six epithelia (EP) networks, three fetal tissue (FE) networks, 14 stromal cell (ST) networks, four visceral cell (VI) networks] and predict seven cell lineage TF interaction networks, respectively. We further analyze the dynamics and modularity of them, identify cell lineage-specific hub TFs and explore the relationship between hub TFs and other general TFs, revealing that hub TFs play a significant role in cell lineage-specific metabolism, development, differentiation, and proliferation. Furthermore, we propose that TFs participating in cell lineage activities are not necessarily specific, while both hub TFs and TF interaction patterns are dynamic, which indicates specific hub TFs exert cellular or lineage-specific regulation by interacting with non-specific TFs. Taken together, our integrative analysis from multiple data can reveal more precise and extensive description about human TF combinatorial interactions.

## Materials and methods

### Data sources and preprocessing: Application I

We extract the bed files of 690 ChIP-seq experiments of 155 human TFs (GRCH37/hg19 assembly) from the ENCODE project (http://www.genome.ucsc.edu/ENCODE/) (Supplementary Table 1). We merge the overlapping sites of different conditions for the same TF by BedTools (Quinlan and Hall, 2010) to generate non-redundant binding regions for each TF in various conditions. The resulting peak scores are defined as an average of the summit of merged peaks. Lastly, we get 155 new bed files of TF binding sites (Griffon et al., 2015). After generating the non-redundant merged peaks for each TF, we adopt the IntervalStats tool (Chikina and Troyanskaya, 2012) to identify TF interaction score for each pair of TFs. Specifically, a pair of TFs consists of a query set and a reference set of binding sites respectively, and for each peak in query set of binding sites, we use IntervalStats to compute a *p*-value, representing a significant degree of overlap with the reference set. A peak is identified as a significant one if *p*-value < 0.05. We repeat this procedure for each peak in query set and calculate the percentage of the significant overlapping peaks between the TF pair. The percentage is defined as the interaction score of this TF pair. Based on this, we obtain an asymmetric matrix of TF interactions (Griffon et al., 2015). Finally, we transform this TF interaction matrix into a symmetric one with the average of two corresponding elements and normalized the symmetric matrix into a range of [0, 1] (see section The Normalization of a Weighted TF Interaction Network).

We also download the gene expression and DNA methylation data of all cell lines profiled by RRBS and RNA-seq respectively from the ENCODE project (Supplementary Tables 2, 3) and extract 100 DNA methylation samples and 61 gene expression samples of all 155 TFs, respectively to infer TF co-expression and co-methylation networks. Specifically, we generate a co-expression network with the absolute value of the Pearson correlation coefficient (PCC). Then, the weighted co-expression network is normalized into a range of [0, 1] (see section The Normalization of a Weighted TF Interaction Network). Meanwhile, for the DNA methylation datasets, we define the region within ±2 kb from a known transcription start site (TSS) based on GENCODE v19 (Harrow et al., 2012) as the promoter of the gene, and compute the methylation level of a gene by an average of beta value between 0 and 1 (ratio of methylated to the sum of methylated and unmethylated sites) of the promoter region. Then we extract the DNA methylation profiles of all 155 TFs from all samples and construct a co-methylation network similarly. Finally, we obtain a weighted co-methylation network and normalize it into a range of [0, 1] (see section The Normalization of a Weighted TF Interaction Network). In summary, we obtain a TF interaction tensor by putting these three networks together, which is used as the input of BCPF (Figure 1).

### Data sources and preprocessing: Application II

We obtain the 41 TF regulatory networks of diverse cell and tissue types derived from DNase I footprints datasets combining with the predicted TRANSFAC motif-binding sites by Neph et al. (2012). All 41 networks include 38,393 unique directed regulatory interactions among 475 TFs with an average of 11,193 ones in each cell type. The 41 cell types is divided into 8 lineages manually in Neph et al. (2012): blood (seven cell types), cancer (two cell types), embryonic stem cells (one cell types), endothelia (four cell types), epithelia (six cell types), fetal tissues (three cell types) stromal cells (14 cell type), and visceral cells (four cell types).

For each TF pair, we determine whether the two TFs occupy the promoter proximal regions together of genes of all TFs (Supplementary Figure 2). We then count times of the two TFs co-occupying in promoters in the same cell type and define the TF interaction score by Jaccard index as

(1) $$\begin{array}{l} \text{Score}\left({\text{TF}}_{\text{i}},{\text{TF}}_{\text{j}}\right)=\frac{\text{Count}\left(\text{Occupy}\left({\text{TF}}_{\text{i}},{\text{TF}}_{\text{j}}\right)\right)}{\text{Count}\left(\text{Occupy}\left({\text{TF}}_{\text{i}}\right)\cup \text{O}\text{ccupy}\left({\text{TF}}_{\text{j}}\right)\right)},\\ \;i,j\in \left[1,N\right], \end{array}$$

where Occupy(TF_i_, TF_j_) denotes the set of genes collaborating to occupy TF promoters by both TF_i_ and TF_i_, Occupy(TF_i_) represents the set of genes occupying TF promoters by *T*F_i_, Count(S) is a counting function of set *S*, *N* is the number of all TF genes. Score (TF_i_, TF_j_) defines the degree of interaction between TF_i_ and TF_j_. Finally, we can get the weighted TF interaction network for each cell type. Thus, we can apply BCPF to all TF interaction networks (form a ternsor) of a cell lineage to infer a cell lineage TF interaction network.

### The normalization of a weighted TF interaction network

The weighted networks are constructed using different types of datasets and different similarity measures. Thus, we use the min-max normalization to transform the weights into a range of [0, 1]. The weight with extremely high/low signals may skew the normalized weight scores. To avoid this, we truncate the weight scores at 95 and 5 percentile and set the weight score of such edges to 1 and 0 respectively (Teng et al., 2014).

### Bayesian CP factorization of incomplete tensors (BCPF)

We introduce the BCPF approach (Zhao et al., 2015) to infer principal multilinear factors of incomplete and noisy tensor with low-rank constraint, each of which contains three vectors and indicates the relative contribution scores of each elements in three dimensions. We can predict the distribution of unknown entries from tensor by the corresponding factor contribution scores of the entry.

The tensor is defined as a *N*th-order tensor of size. *I*_1_ × *I*_2_ × ⋯ × *I*_*N*_. In this model, a binary tensor O of the same size with Y is given as an indicator tensor of observed entries. Y is assumed as a noisy observation of true latent tensor, that is, Y = X + ε, where ε is a noisy term following i.i.d. Gaussian distribution, i.e., $\varepsilon \sim \prod_{i_{1},\cdots \;,i_{N}}N\left(0,\tau^{-1}\right)$. The true latent tensor is generated by the CP model, which is defined as

(2) $$\begin{array}{l} X=\overset{R}{\underset{r=1}{\sum }}{\text{a}}_{r}^{\left(1\right)}◦\cdots ◦{\text{a}}_{r}^{\left(N\right)}, \end{array}$$

where ◦ denotes the outer product of vectors. X is factorized as a sum of *R* rank-one tensors by the CP factorization. The set of factor matrices is represented by ${\left\{{\text{A}}^{\left(n\right)}\right\}}_{n=1}^{N}$, where the mode-n factor matrix ${\text{A}}^{\left(n\right)}\in {\mathbb{R}}^{I_{n}\times R}$ can be denoted as row-wise or column-wise vectors,

(3) $$\begin{array}{l} {\text{A}}^{\left(n\right)}={\left[{\text{a}}_{1}^{\left(n\right)},\ldots ,{\text{a}}_{i_{n}}^{\left(n\right)},\ldots ,{\text{a}}_{I_{n}}^{\left(n\right)}\right]}^{T}=\left[{\text{a}}_{\cdot 1}^{\left(n\right)},\ldots ,{\text{a}}_{\cdot r}^{\left(n\right)},\ldots ,{\text{a}}_{\cdot R}^{\left(n\right)}\right]. \end{array}$$

The likelihood of the CP generative model with noise assumption, is factorized over observed tensor elements:

(4) $$\begin{array}{l} p\left(Y_{\Omega }|{\left\{{\text{A}}^{\left(n\right)}\right\}}_{n=1}^{N},\tau \right)=\\ \overset{I_{1}}{\underset{i_{1}=1}{\prod }}\ldots \overset{I_{N}}{\underset{i_{N}=1}{\prod }}N{\left(Y_{i_{1}i_{2}\ldots i_{N}}|\langle {\text{a}}_{i_{1}}^{\left(1\right)},{\text{a}}_{i_{2}}^{\left(2\right)},\cdots \;,{\text{a}}_{i_{N}}^{\left(N\right)}\rangle ,\tau^{-1}\right)}^{O_{i_{1}i_{2}\ldots i_{N}}}, \end{array}$$

where τ denotes the noise precision, $\langle {\text{a}}_{i_{1}}^{\left(1\right)},{\text{a}}_{i_{2}}^{\left(2\right)},\cdots \;,{\text{a}}_{i_{N}}^{\left(N\right)}\rangle$ is a generalized inner-product of *N*-vectors and Y_*i*_1_*i*_2_…*i*_*N*__ is generated from multiple *R*-dimension latent vector $\left\{{\text{a}}_{i_{n}}^{\left(n\right)}|n=1,\ldots ,N\right\}$, in which multilinear interaction structure is taken into account.

Meanwhile, to infer effective dimensionality of the latent space (i.e., *R*) and avoid overfitting, all model parameters are considered as latent variables and a sparsity-inducing prior is specified with shared hyperparameters. For each mode-*n* factor matrix, a prior distribution over the latent factors is specified, which is governed by hyperparameters **λ** = [λ_1_, …, λ_*R*_], where λ_*r*_ corresponds to *r*-th component in **A**^(*n*)^, which is

(5) $$\begin{array}{l} p\left({\text{A}}^{\left(n\right)}|\lambda \right)=\overset{I_{n}}{\underset{i_{n}=1}{\prod }}N\left({\text{a}}_{i_{n}}^{\left(n\right)}|0,\Lambda^{-1}\right),\forall n\in \left[1,N\right], \end{array}$$

where Λ = *diag*(λ) is known as the precision matrix, which is shared by latent factor matrices in all modes. Next, the further definition of hyperprior over λ is given as follows

(6) $$\begin{array}{l} p\left(\lambda \right)=\overset{R}{\underset{r=1}{\prod }}G\text{a}\left(\lambda_{r}|c_{0}^{r},d_{0}^{r}\right), \end{array}$$

where a Gamma distribution is defined as $Ga\left(x|a,b\right)=\;\frac{b^{a}x^{a-1}e^{-bx}}{\Gamma \left(a\right)}$.

The dimensionality of latent space (i.e., *R*) is initialized to be a maximum possible value and the effective dimensionality can be inferred automatically from observed data by a Bayesian inference framework.

To complete the model, a hyperprior over the noise precision is specified by

(7) $$\begin{array}{l} p\left(\tau \right)=Ga\left(\tau |a_{0},b_{0}\right). \end{array}$$

To simplify notation, all unknown variables are collected and denoted as $\Theta =\left\{A^{\left(n\right)},\ldots ,A^{\left(n\right)},\lambda ,\tau \right\}$. The joint distribution is written as:

(8) $$\begin{array}{l} p\left(Y_{\Omega },\Theta \right)=p\left(Y_{\Omega }|{\left\{{\text{A}}^{\left(n\right)}\right\}}_{n=1}^{N},\tau \right)\overset{N}{\underset{n=1}{\prod }}p\left({\text{A}}^{\left(n\right)}|\lambda \right)p\left(\lambda \right)p\left(\tau \right). \end{array}$$

Generally, maximum a posteriori (MAP) estimation of **Θ**. can be achieved by optimizing (Equation 8). In contrast to the point estimation, our objective is to develop a Bayesian inference method to compute the full posteriori distribution of all variables in **Θ**, which is computed by

(9) $$\begin{array}{l} p\left(\Theta |Y_{\Omega }\right)=\frac{p\left(\Theta ,Y_{\Omega }\right)}{\int p\left(\Theta ,Y_{\Omega }\right)d\Theta }. \end{array}$$

However, exact inference in Equation (8) is obviously analytically intractable. A deterministic approximate inference under variational Bayesian framework is developed to learn a probabilistic CP factorization model. Specifically, a distribution *q*(**Θ**) is an approximation of true posteriori distribution *p*(Y_Ω_|**Θ**) by minimizing the KL divergence, that is,

(10) $$\begin{array}{l} \text{KL}\left(q\left(\Theta \right)||p\left(\Theta |Y_{\Omega }\right)\right)=\int q\left(\Theta \right)\left\{\frac{q\left(\Theta \right)}{p\left(\Theta |Y_{\Omega }\right)}\right\}d\Theta \\ =\;lnp\left(Y_{\Omega }\right)-\int q\left(\Theta \right)\left\{\frac{p\left(Y_{\Omega },\Theta \right)}{q\left(\Theta \right)}\right\}d\Theta , \end{array}$$

where *lnp*(Y_Ω_) is a constant, the maximum of the lower bound $L\left(q\right)=\int q\left(\Theta \right)\left\{\frac{p\left(Y_{\Omega },\Theta \right)}{q\left(\Theta \right)}\right\}d\Theta$ occurs when the KL divergence is close to zero.

According to the mean-field theory, it is assumed that *q*(**Θ**) can be factorized as

(11) $$\begin{array}{l} q\left(\Theta \right)=q\left(\lambda \right)q_{\tau }\left(\tau \right)\overset{N}{\underset{n=1}{\prod }}q_{n}\left({\text{A}}^{\left(n\right)}\right). \end{array}$$

By maximizing the lower bound L(q), the functional forms of *q*_*j*_(**Θ**_*j*_) can be explicitly deduced over all variables except **Θ**_*j*_ in turn, and the form of the *j*-th factor is given by

(12) $$\begin{array}{l} \ln\;q_{j}\left(\Theta_{j}\right)={𝔼}_{q_{j}\left(\Theta \backslash \Theta_{j}\right)}\left[\ln\;p\left(Y_{\Omega },\Theta \right)\right]+\text{const}, \end{array}$$

where 𝔼_*q*_*j*_(**Θ**\**Θ**_*j*_)_[·] represents the expectation with regard to the distribution over all variables except **Θ**_*j*_. Due to all distributions are from exponential family and conjugate with their parents distribution, we can get a closed-form update rule for the elements of **Θ**.

More specifically, the entire process of model inference is as below. Frist, we specify the initialization of model parameters, including **a**, **b**, *c, d*, the maximum rank *R* and ${\left\{{\text{A}}^{\left(n\right)}\right\}}_{n=1}^{N}$. Second, we iterate to update λ and τ which results in a new prior over $\left\{{\text{A}}^{\left(n\right)}\right\}$, and the new $\left\{{\text{A}}^{\left(n\right)}\right\}$ will affect λ, τ in turn. Third, the tensor rank *R* can be updated by the non-zero components of the factor matrices. Fourth, we iterate to perform the above two steps until convergence. Finally, we compute the predictive distribution of all entries.

### Identification of strongly relevant interactions for each TF

To detect strong interactions among TFs, for each TF, we identify a list of interactions, whose weights exceed α times the interquartile range above the 75th percentile of all edge weights associated with this TF (Griffon et al., 2015) (Supplementary Figure 1). Finally, we gain a weighted network consisting of all strongly relevant interactions.

### Two gold standard sets of TF interactions

We build the first set of gold standard TF interactions verified by experiments from HumanNet (Lee et al., 2011), iRefIndex (Razick et al., 2008), and a large-scale two-hybrid mapping study (Ravasi et al., 2010). We think that a predicted TF interaction is true if it appears in the gold standard set for the first application.

We normalized 41 weighted networks into a range of [0, 1] as above. For each TF pair, we average the score of each TF pair of all 41 cell types except missing entries and define the average as gold standard score of the TF pair. Thus, we obtain the second gold standard TF interaction network. We think that a predicted TF interaction is true if it appears in the gold standard set for both two applications.

### The averaged integrative networks and completing cell type TF networks

To better show the accuracy of cell lineage networks (CL-Nets) generated by BCPF, we first generate seven simple and rough TF interaction networks by computing the average of existing edges in cell types of the same lineage respectively, and then we define these networks as the averaged integrative networks (AI-Nets). Next, due to the existence of missing entries in cell type TF interaction networks (CT-Nets), we complete missing values based on the interactions of the AI-Nets of the corresponding cell lineage.

## Results

### Application I: inferring TF combinatorial interactions from multiple ENCODE datasets

**Figure 2:**
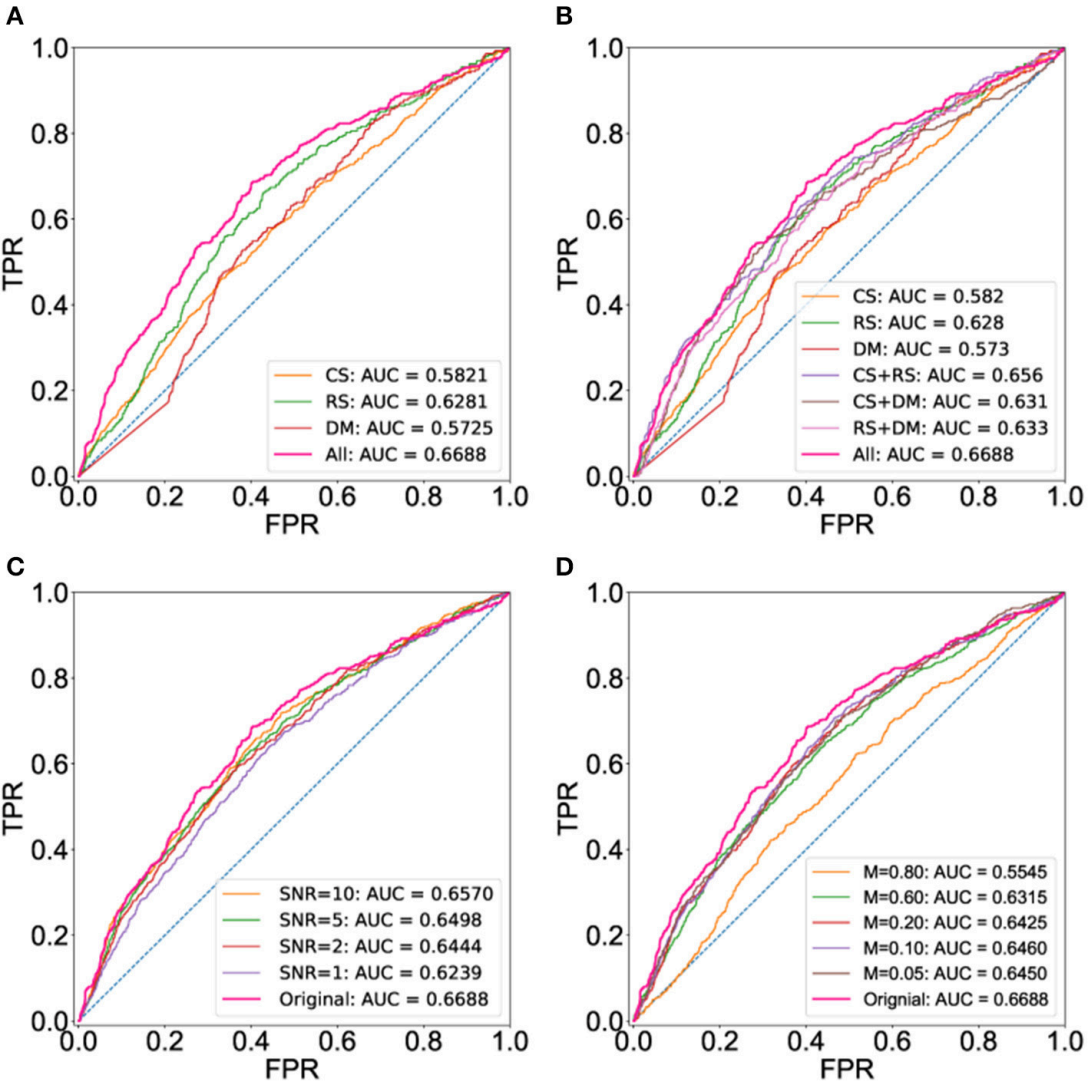
ROC curves using the set of gold standard TF interactions based on physical protein-protein interactions. ROC curves are computed using a set of gold interactions from HumanNet, iRefIndex and a large-scale two hybrid mapping study. **(A)** ROC curves of the integrated and three individual networks. **(B)** ROC curves of the integrated networks, three individual ones and integrated ones with any two datasets. **(C)** ROC curves of integrated network with different levels of noise. **(D)** ROC curves of integrated network with different percentage of missing entries.

Here, we apply BCPF to integrate individual networks including co-binding, co-expression and co-methylation networks to obtain a global weighted TF interaction network. We also obtain the integrated networks with any two of the three networks using BCPF. To assess the functional relevance of the integrated network, we compare the prediction accuracy of the integrated network with the three individual networks and integrated ones with any two datasets based on the first gold standard set in terms of ROCs. As we expected that the integrated network has a higher area under curve (AUC) than any individual networks (Figure 2A) and integrated networks with any two datasets (Figure 2B). Meanwhile, we also test the robustness of BCPF by removing diverse percentage of entries or adding diverse degree of Gaussian noise to the original individual networks, respectively. We can see that BCPF is very effective to recover the missing entries or original signals (Figures 2C,D).

**Figure 3:**
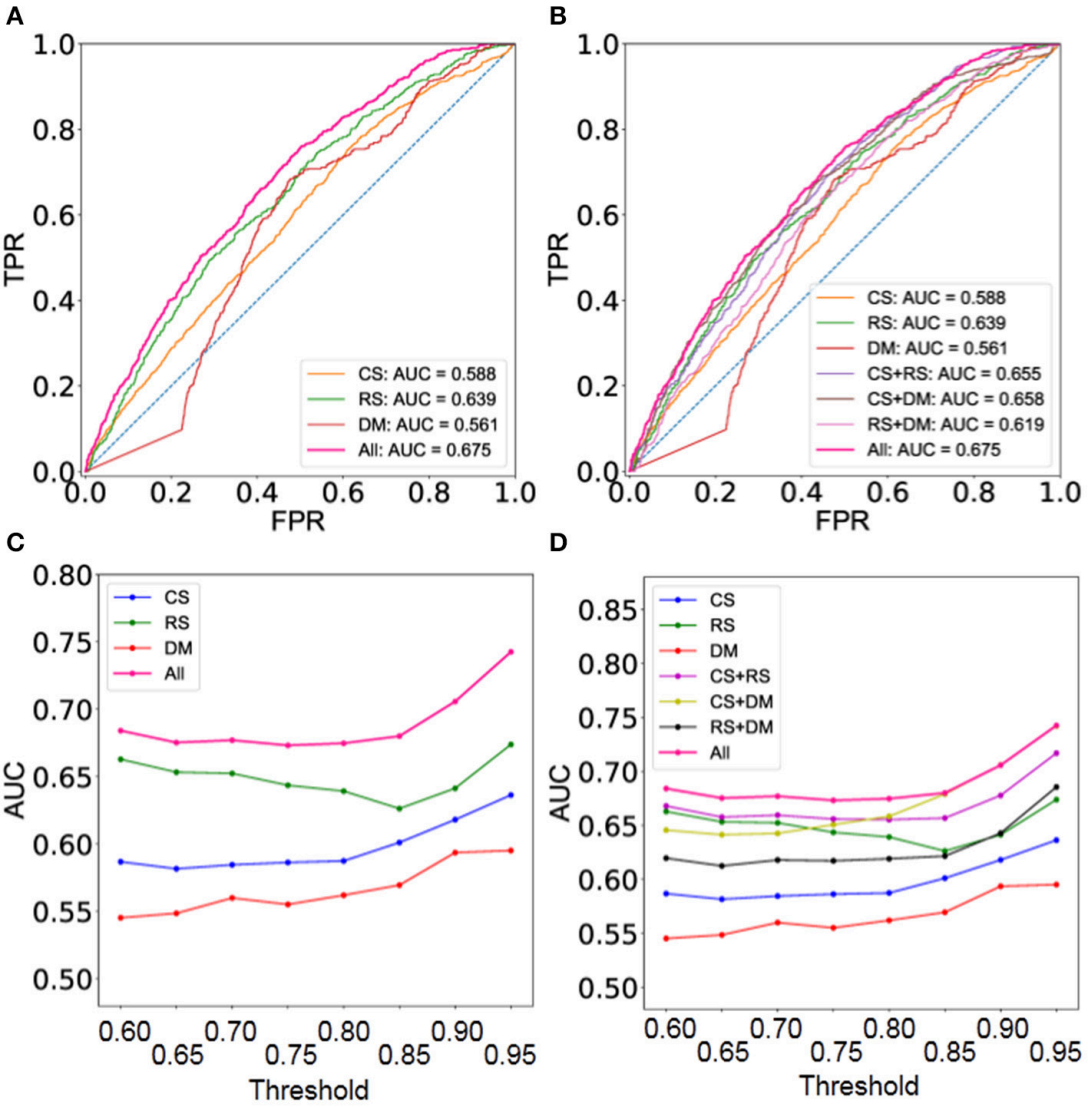
Performance evaluation using the set of gold standard TF interactions based on 41 cell type TF regulatory networks. **(A)** ROC curves of the integrated and three individual networks at threshold = 0.8. **(B)** ROC curves of the integrated networks, three individual ones and integrated ones with any two datasets at threshold = 0.8. **(C)** AUC of the integrated and three individual networks at different thresholds. **(D)** AUC of the integrated networks, three individual ones and integrated ones with any two datasets at different thresholds.

Moreover, the integrated network predicted by BCPF obtains significant improvements at different thresholds in terms of ROCs with respect to the second gold standard TF interaction network (Figure 3A and Supplementary Figure 3). Figure 3C offers a detailed description of accuracy of the integrated network in terms of AUCs (Supplementary Table 4). Generally, the integrated network with all three networks shows better accuracy than those of any two networks at different thresholds (Figures 3B,D and Supplementary Figure 4).

Based on the weighted TF interaction network derived by BCPF from multiple ENCODE datasets, we identify 81 significant TF interactions with α = 1.5 (Materials and Methods), which includes 38 *de novo* ones with respect to all individual networks (Supplementary Figure 5 and Supplementary Table 5). Interestingly, we find that these TFs show significant biological relevance through literature-mining. For example, Mohammed et al. (2013) developed an approach RIME and detected 108 co-factors of ESR1 in breast cancer cells. Integrated network identifies GATA3, FOXA1, HDAC2, and ZNF217 interacting with ESR1, while only the last two ones appear in the three individual networks. That HDAC8 deacetylates ESR1 and increases ESR1 DNA-binding activity confirms our prediction (Wilson et al., 2010; Chakrabarti et al., 2015). Furthermore, previous studies have confirmed a high degree of co-occupancy among BRF1, BDP1, and RPC155 (Canella et al., 2010; Moqtaderi et al., 2010) whose interactions are predicted in the integrated network, suggesting the capability of integrative analysis with BCPF. In addition, RXRA is detected as a hub node interacting with FOXA1, FOXA2, HNF4A, and HNF4G in the integrated network, but not in the individual networks. Interestingly, HNF4G implies a negative regulation by interacting with FOXA1, RXRA, and HNF4A in the human hepatoma cell line and some studies have showed that interrelationships among FOXA1, FOXA2, HNF4A, and HNF4G affect transcriptional regulation (Tomaru et al., 2009). Moreover, AP-1 family mediates gene regulation in response to many extracellular stimuli which consists of JUN, FOS and ATF families. Surprisingly, all factors of the module (JUN, JUNB, FOSL1, and ATF3) identified only by the integrated network are from AP-1 family and AP-1 subunits JUN and JUNB have been shown to act antagonistically to control cell transformation, differentiation, and expression of AP-1 dependent target genes (Szabowski et al., 2000; Shaulian and Karin, 2002; Hess et al., 2004). The most intriguing finding is that the module (BATF, IRF4, RUNX3, EBF1, PAX5, BCL11A, POU2F2, MEF2A, and MEF2C) we identified reveals strong associations with B and T cells. This can be seen by the following observations including (1) the co-binding of BATF, IRF4, and RUNX is essential for the efficient transformation of human primary B lymphocytes cell lines (Saha et al., 2011; Jiang et al., 2014); (2) BATF-IRF interactions mediate compensatory dendritic cell development and function in T and B cells (Tussiwand et al., 2012); (3) Combined heterozygous loss of EBF1 and PAX5 allows for T-lineage conversion of B cell progenitors (Ungerbäck et al., 2015); (4) B cell-specific enhancers are enriched for binding sites of BATF, IRF4, EBF, PAX5, EBF, and PU.1, all of which are known to be specific regulators in B cells (Teng et al., 2011), and the interaction of PU.1 and POU2F2 regulate human U2 snRNA gene expression (Ström et al., 1996); (5) MEF2 (MEF2A/MEF2C) proteins cooperate with the products of gene IRF4 to induce waves of transcriptional regulation and promote early B-cell development (Herglotz et al., 2016). Meanwhile, Analysis using CaGe (http://mgrc.kribb.re.kr/cage/) shows this module is closely related with blood tissue (Supplementary Table 6). Those observations suggest that this module tends to be a true functional unit for blood cell lineage. In addition to these known interactions, the analysis of other novel interactions such as the module (MYC, JUN, TFAP2A, BACH1) and RELA-BCL3 interactions also offer new insights into the mechanisms of TF regulations. In summary, integrative analysis of multiple ENCODE datasets provides a global atlas of TF transcriptional regulation and elucidate many *de novo* and significant TF interactions for further studies.

### Application II: inferring cell lineage TF interactional networks based on cell type TF regulatory networks

**Figure 4:**
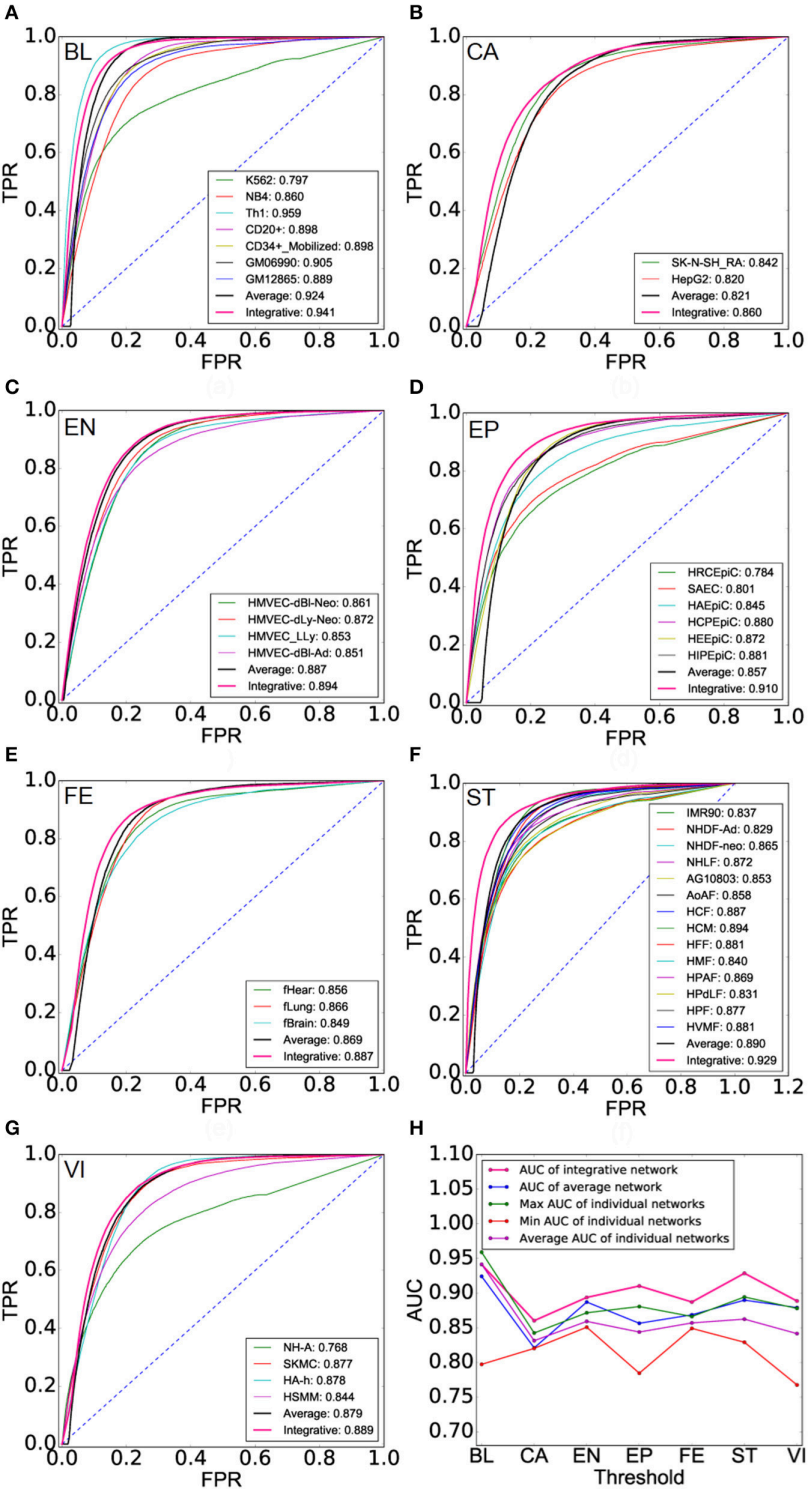
Performance evaluation of cell lineage TF interaction networks. **(A–G)** ROCs of cell lineage networks (CL-Nets), average-integrative-networks (AI-Nets), and cell type TF interaction networks (CT-Nets) of the corresponding cell lineage across all seven cell lineages. **(H)** AUC of CL-Nets, AI-Nets as well as the maximum, minimum and average AUC of CT-Nets in the same cell lineage across all seven cell lineages.

We expect to recover the underlying landscape of cell lineage TF interactions by integrating multiple cell type TF interactions of a cell lineage. We can see that cell lineage networks get a higher accuracy than AI-Nets and cell type TF networks of this cell lineage with respect to the second gold standard TF interaction network, demonstrating the integrative analysis gain additional information (Figures 4A–G and Supplementary Table 7). Finally, we list the AUC of CL-Nets, AI-Nets, as well as the maximum, minimum and average AUC of CT-Nets of the corresponding cell lineage for each cell lineage respectively (Supplementary Table 8). CL-Nets have the highest AUC in all cases except blood lineage (Figure 4H).

**Figure 5:**
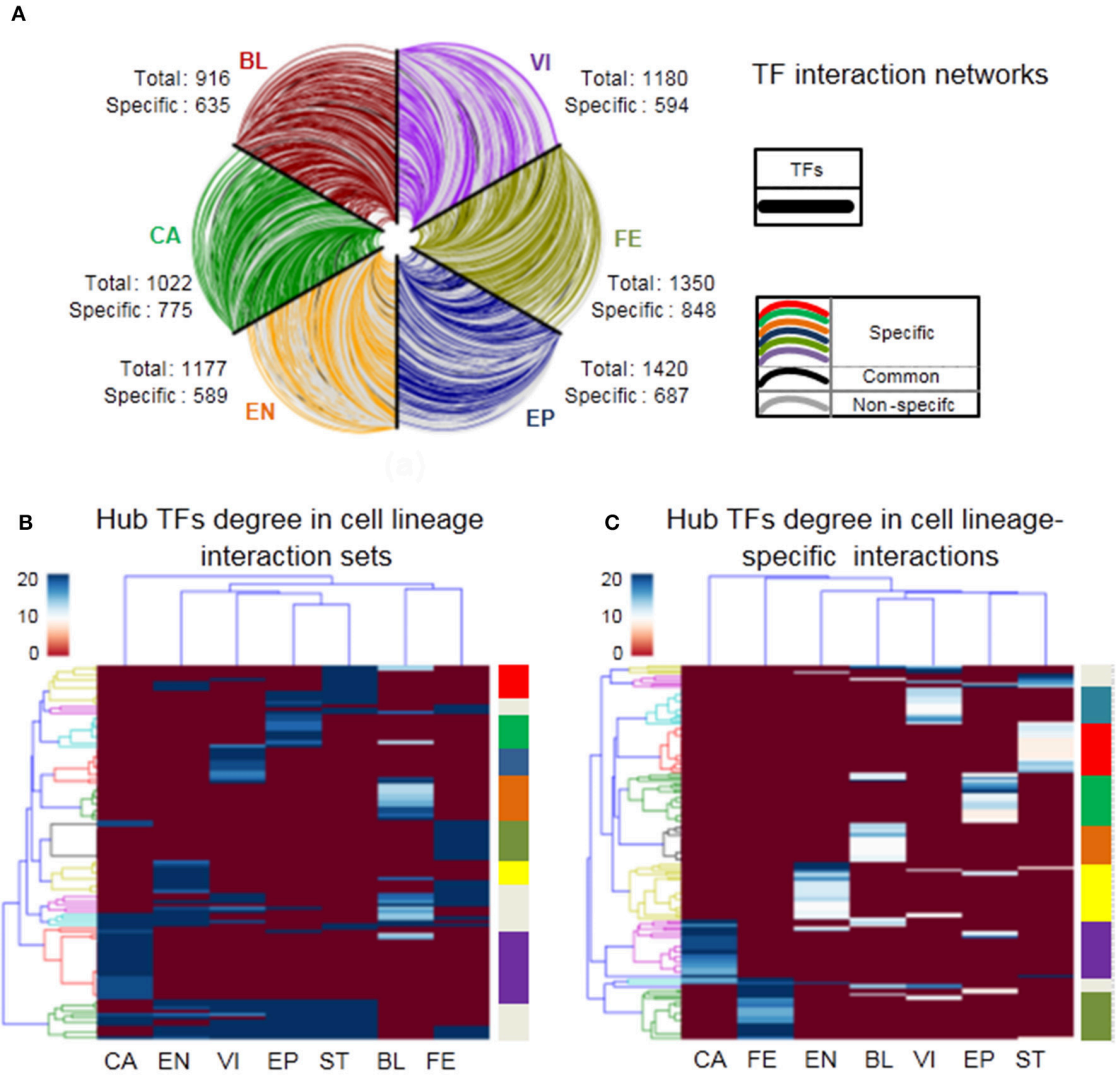
Characteristics of cell lineage networks. **(A)** TF interactions among all TFs of six cell lineages. All TFs are similarly ordered in each axis, cell lineage-specific edges are labeled by different colors in the legend, edges occurred in two or more cell lineages are colored gray, and edges occurred in all cell lineages are colored black. **(B,C)** Heatmap showing hierarchical clustering of hub TFs degree (top 5%) in cell lineage interactionsand cell lineage-specific interactions respectively, where branch colors of the y-axis represent which classes there TF nodes belong to.

We next explore the dynamics and modularity of cell lineage TF interactions. First, we predict 916, 1,022, 1,177, 1,420, 1,350, 1,449, 1,180 strongly relevant interactions from seven cell lineages: BL, CA, EN, EP, FE, ST, and VI, respectively (Materials and Methods). Furthermore, we identify 635 (69%), 775 (76%), 589 (50%), 687 (48%), 848 (62%), 612 (42%), 594 (50%) cell lineage-specific TF interactions (only found in one cell lineage) respectively and only 29 (2%) HK TF interactions (found in all cell lineages). It suggests that specific TF interaction pairs are fairly common across the lineages with an average ratio 57% of all strong interactions (Supplementary Table 9). Moreover, cell lineage-specific interaction networks show a scale-free distribution, that is, there are a large number of low degree TFs and a small number of hub TFs (Supplementary Figure 6). Next, we plot the strong interaction relationships among all TFs participating in lineage activities for six different cell lineages (Figure 5A), which reveals the dynamics and specificity of TF interactions.

To further investigate the mechanism of cell lineage specificity in depth, we explore the classifications of cell lineages using hub TFs from cell lineage interaction sets and cell lineage-specific interactions (the top 5% of TFs with the largest degrees). For cell lineage interaction sets, Figure 5B shows that 113 hub genes tend to be divided into 10 different clusters (Supplementary Table 10). 7/10 clusters are obviously related to 7 corresponding cell lineages, which may be functionally related to specific lineage identity. 3/10 clusters regulate at least one cell lineage. 83/113 (72%) are cell lineage-specific TFs. For cell lineage-specific interactions, note that 163 hub TFs tend to be divided into 9 different sets that include seven sets (148 TFs) enriched in seven specific cell lineages respectively and only two sets (15 TFs) relates to multiple cell lineages (Figure 5C and Supplementary Table 10). It suggests that different cell lineages tend to be regulated by specific hub TFs (91% of all hub TFs). We next list the number of hub TFs in Table 1 and the corresponding details between hub TFs and cell lineages in Supplementary Table 10. Each lineage is affected by 7~24 hub TFs in the cell lineage interaction and cell lineage-specific interaction networks, respectively (Table 1).

**Table 1:** The number of hub TFs for all cell lineages.

**Lineages**	**BL**	**CA**	**EN**	**EP**	**FE**	**ST**	**VI**
#A-sets	14	22	7	10	12	11	8
#B-sets	16	25	25	22	21	22	16

*#A-sets, The number of hub TFs for cell lineage interactions; #B-sets, The number of hub TFs for cell lineage-specific interactions*.

To illustrate the biological functions of hub TFs, we take cancer lineage (including liver cancer and neuroblastorma) and blood lineage (including seven cell types) as examples in the following.

For cancer cell lineage, we detect 30 hub TFs related with cancers from these two sets together. Seven TFs (FOXA1, FOXA2, RARG, STAT4, MEF2C, SOX18, and GXS2) have been identified to be likely to affect the metabolism, development, differentiation and proliferation of liver cancer in previous studies. For example, Li et al. have uncovered that the winged helix transcription factors FOXA1 and FOXA2 play a central role in controlling estrogen and androgen of the liver by interacting with ERα and AR, explaining the sexual dimorphism of liver cancer in mammals (Li et al., 2012). Moreover, alterations in the expression or activity of specific retinoic acid receptors (RARA, RARB, and RARG) were well-known TFs controlling transcription and inducing diverse types of cancer by recruiting different co-regulator complexes. Khetchoumian et al. reported that RARA regulates liver cancer in an antagonistic manner by acting together with Trim24 (formerly known as TIF1α) and suggested aberrant activation of RARA is deleterious to liver homeostasis (Khetchoumian et al., 2007). Bai et al. explored the roles of MEF2C in regulating liver progression by forming complexes with β-catenin (Bai et al., 2015). Jiang et al. found significantly lower expression of STAT4 and Wang et al. found overexpression of SOX18 in liver cancer compared with adjacent non-tumor tissues (Jiang et al., 2013; Wang et al., 2015). We also identified 25 hub TFs for brain cancer. At least five of them (ARNT2, NEUROD1, NR1I2, HOXC9, NR4A2) are associated with brain cancer in previous studies. For instance, in brain tumor cell lines, ARNT2 can form functional HIF complexes by co-binding with other factors controlling hypoxic responses of the human EPO enhancer (Stolze et al., 2002). Huang et al. reported that the neuronal differentiation factor (NEUROD1) plays a critical role in the formation of tumor and is functionally related with the neuronal repellent factor Slit2 in neuroblastorma (Huang et al., 2011). Misawa et al. discovered that nuclear receptor 1I2 (NR1I2) regulates promoter activity and can serve as a prognostic marker for neuroblastorma whose expression was decreased in tumors of neuroblastorma by hypermethylation of a CpG island (Misawa et al., 2005). Kocak et al. determined that HOX gene expression patterns are associated with prognostic markers and outcome in neuroblastorma. They also found HOXC9 re-expression triggers neuroblastorma growth and programs cell death (Kocak et al., 2013).

For blood cell lineage, we have uncovered 25 hub factors relating to blood tissue, including 10 known regulators deducing the development and proliferation of cell. For instance, dysregulation of HOX gene family members play a dominant role in mechanism of leukemic transformation (Ferrando et al., 2003) and those co-factor MEIS1 quantitatively regulates the differentiation, cycling activity and self-renewal of MLL leukemia cell (Wong et al., 2007). Moreover, RUNX1 is a key factor regulating a broad spectrum of genes of the myeloid and lymphoid lineages which affects B- and T-cell maturation and dramatically inhibits common lymphocyte progenitor generation. Moreover, RUNX1 is required for definitive hematopoiesis, yet its loss of function is associated with leukemias (Growney et al., 2005).

In addition to these known TFs previously, our analysis reveal some factors with unrecognized functions in specific cell lines. For examples, the dynamic expression of RARB, RARG similar with RARA can control the development and proliferation of liver cancer and HOXB8 from HOX family can play a prominent role as a brain tissue biomarker. Meanwhile, some studies have reported the specific function of NKX3-1, ALX4, and FOXF1 in blood cell types, which are associated with development and proliferation of blood cell lineage.

Together, the above results show that TF interactions participated in lineage activities are dynamic, while TFs forming TF interaction network are not necessarily specific. Moreover, we find hub TFs are specific for driving related lineage activities and determining lineage identity. It suggests that specific hub TFs can play an important role in the ability of transcriptional regulation and the determination of cell identity by interacting with other non-hub TFs.

## Discussion

The accumulation of ChIP-seq provides a detailed description about annotation of TF binding sites and creates great opportunities to discover precise TF complexes. However, it is still difficult to uncover a global and accurate map of regulatory elements due to technique limitation and data noise. To this end, we adopt a powerful method BCPF to integrate multiple datasets to identify a global map of combinatorial regulations. We demonstrate its effectiveness in two applications.

The limitations of this paper are as followings. First, it does not collect multiple ENCODE datasets such as ChIP-seq, RNA-seq, DNA methylation from individual experiment of the same cellular state, which hinders the study of cell-specific TF combinatorial regulation. The accumulation of high-throughput sequencing datasets will give opportunities to integrate multiple omics data and analyze the dynamic of TF interactions across cell lines. Second, we identify individual TFs or TF pairs contributing to cell lineage specificity, however, transcriptional complexes are more likely to exert real functions in living cellular activities intuitively. Those findings give us some hint to explore transcriptional complexes regulating gene expression. Third, the success of techniques exploring chromatin interactions such as Hi-C and ChIA-PET provides a chance for finding distal regulatory elements and inferring binding or non-binding co-factors (Djekidel et al., 2015). Combining chromatin interactions and sequencing datasets in various cell lines will depict a global landscape of TF combinatorial interactions regulating cell-specific genes.

## Author contributions

YY performed the experiments. YY and SZ carried out bioinformatic and statistical analysis. YY, LG, and SZ designed the study, interpreted the results and written the manuscript. All authors read and approved the final manuscript.

### Conflict of interest statement

The authors declare that the research was conducted in the absence of any commercial or financial relationships that could be construed as a potential conflict of interest.
