# Supplementary material for: Integrative Analysis of Transcription Factor Combinatorial Interactions Using a Bayesian Tensor Factorization Approach
Source: Front Genet. 2017 Sep 28;8:140. doi: 10.3389/fgene.2017.00140 (PMC5625019; doi:10.3389/fgene.2017.00140)
Supplement: Supplementary file 2 [file Image1.PDF]

Supplementary Materials

Integrative analysis of transcription factor combinatorial interactions using a Bayesian tensor factorization approach

Yusen Ye [1], Lin Gao [1*], Shihua Zhang [2,3*]*

Correspondence: Lin Gao: lgao@mail.xidian.edu.cn; Shihua Zhang: zsh@amss.ac.cn

1 Supplementary Figures and Tables

1.1 Supplementary Figures

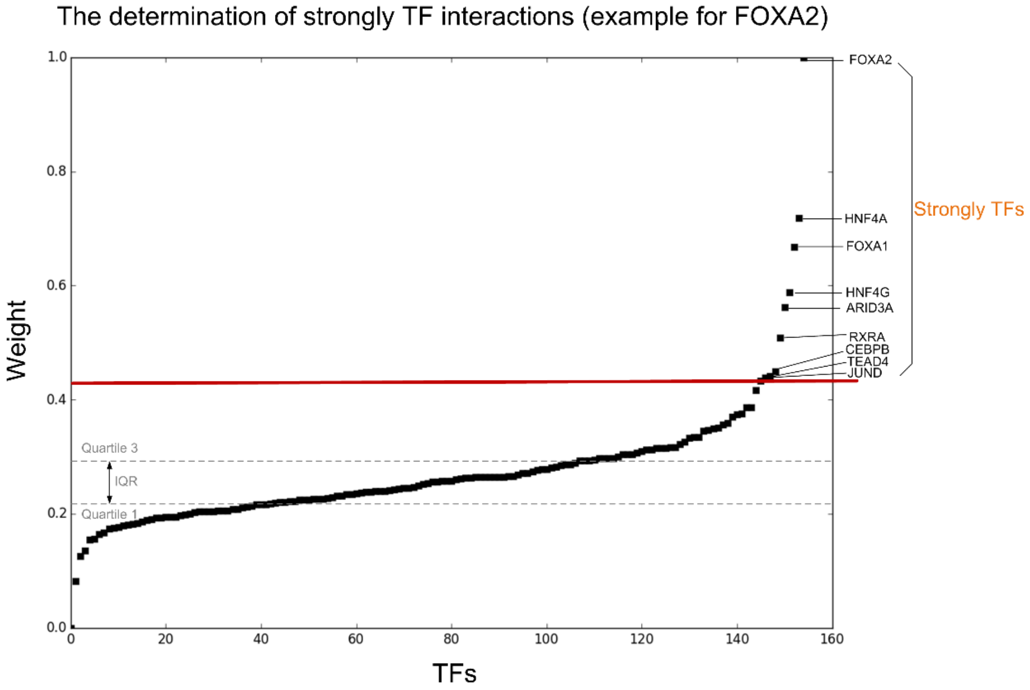

Supplementary Figure 1. The determination of strongly specific TF interactions (example for FOXA1).

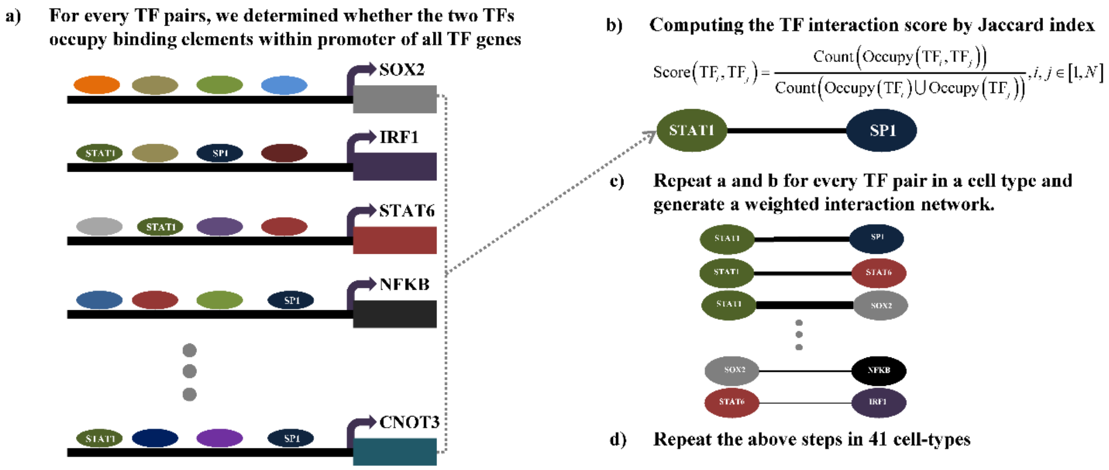

Supplementary Figure 2. Schematic of extraction of cell-specific TF interactions from 41 TF regulatory networks.

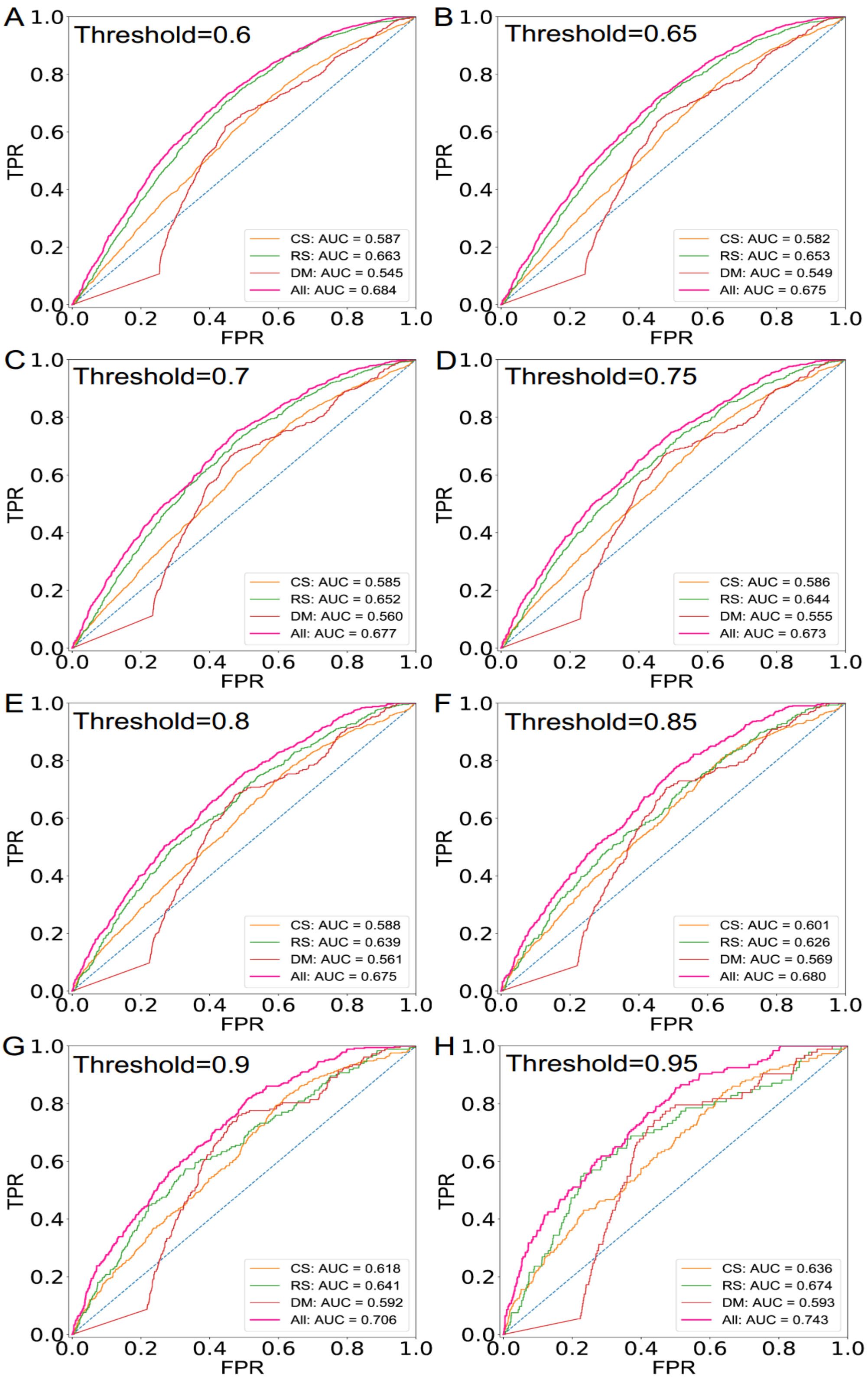

Supplementary Figure 3. ROC curves of the integrated and three individual networks in different thresholds using the set of gold standard TF interactions based on 41 cell TF regulatory networks.

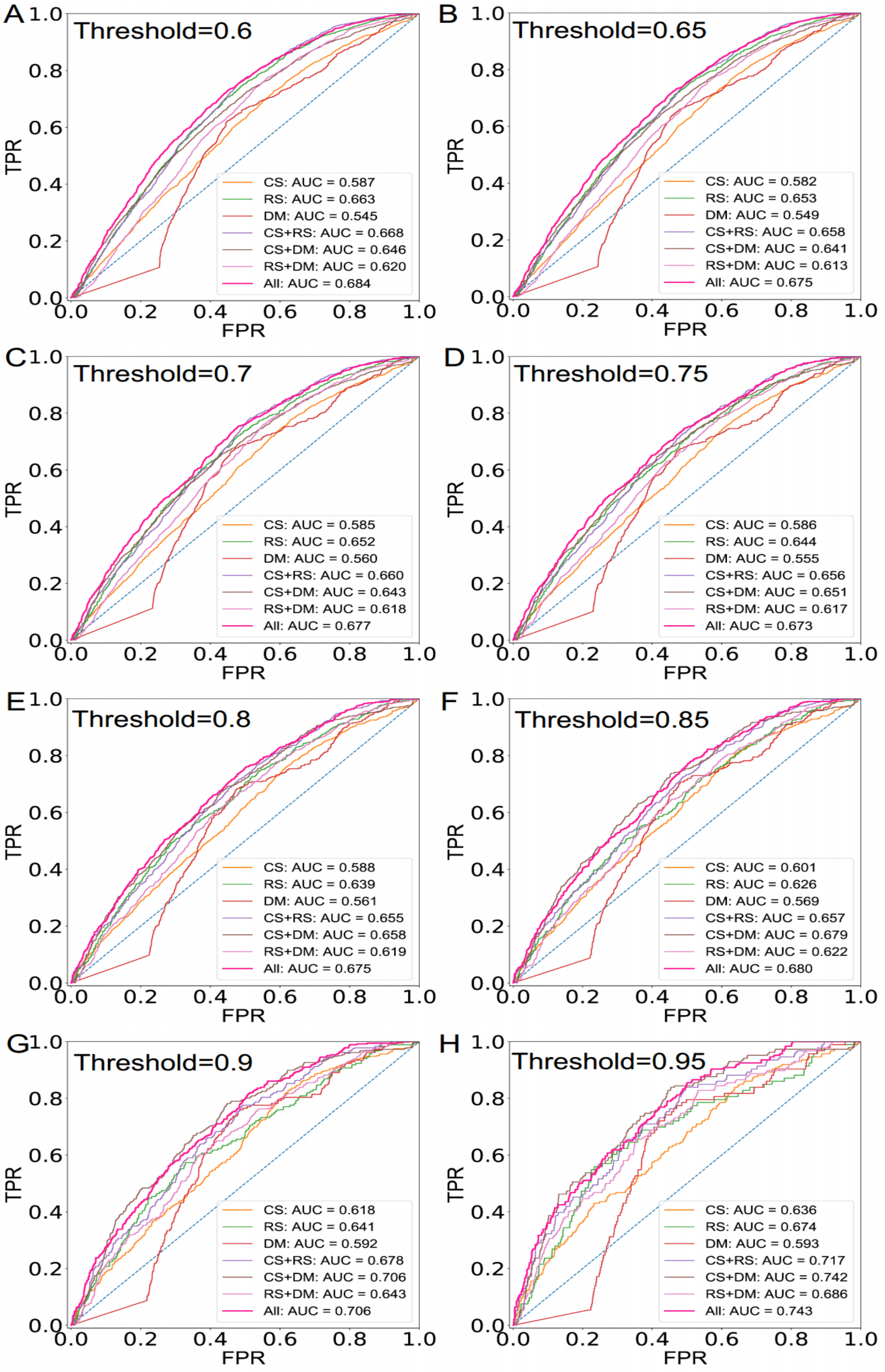

Supplementary Figure 4. ROC curves of the integrated networks, three individual ones and integrated ones with any two datasets in different thresholds using the set of gold standard TF interactions based on 41 cell-specific TF regulatory networks.

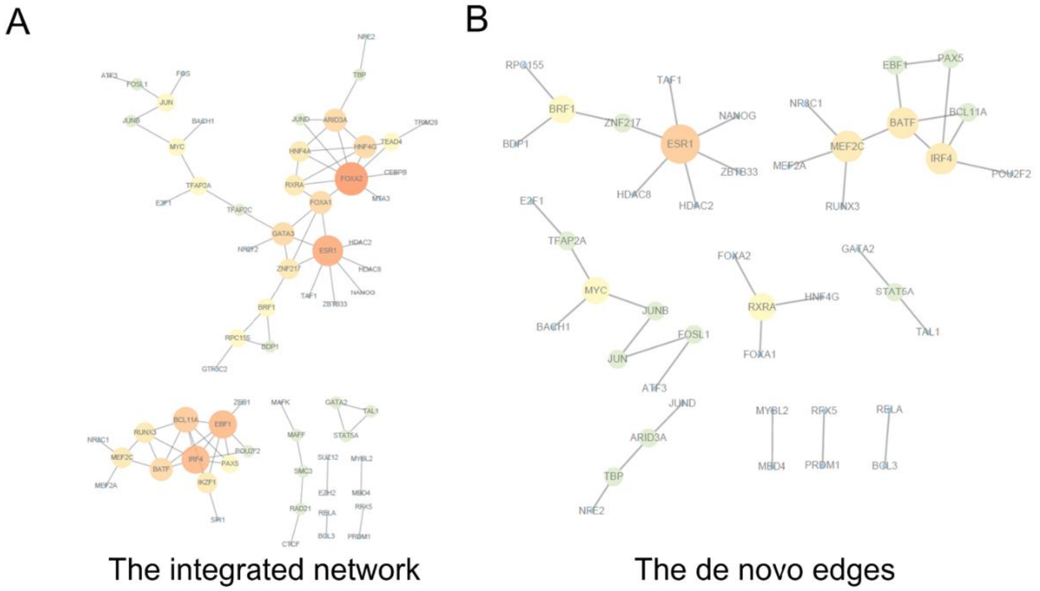

Supplementary Figure 5. Visualization of TF interaction integrated network (A) and *de novo* TF interactions (B). Cytoscape tool is used to visualize 81 significant edges and 38 *de novo* edges.

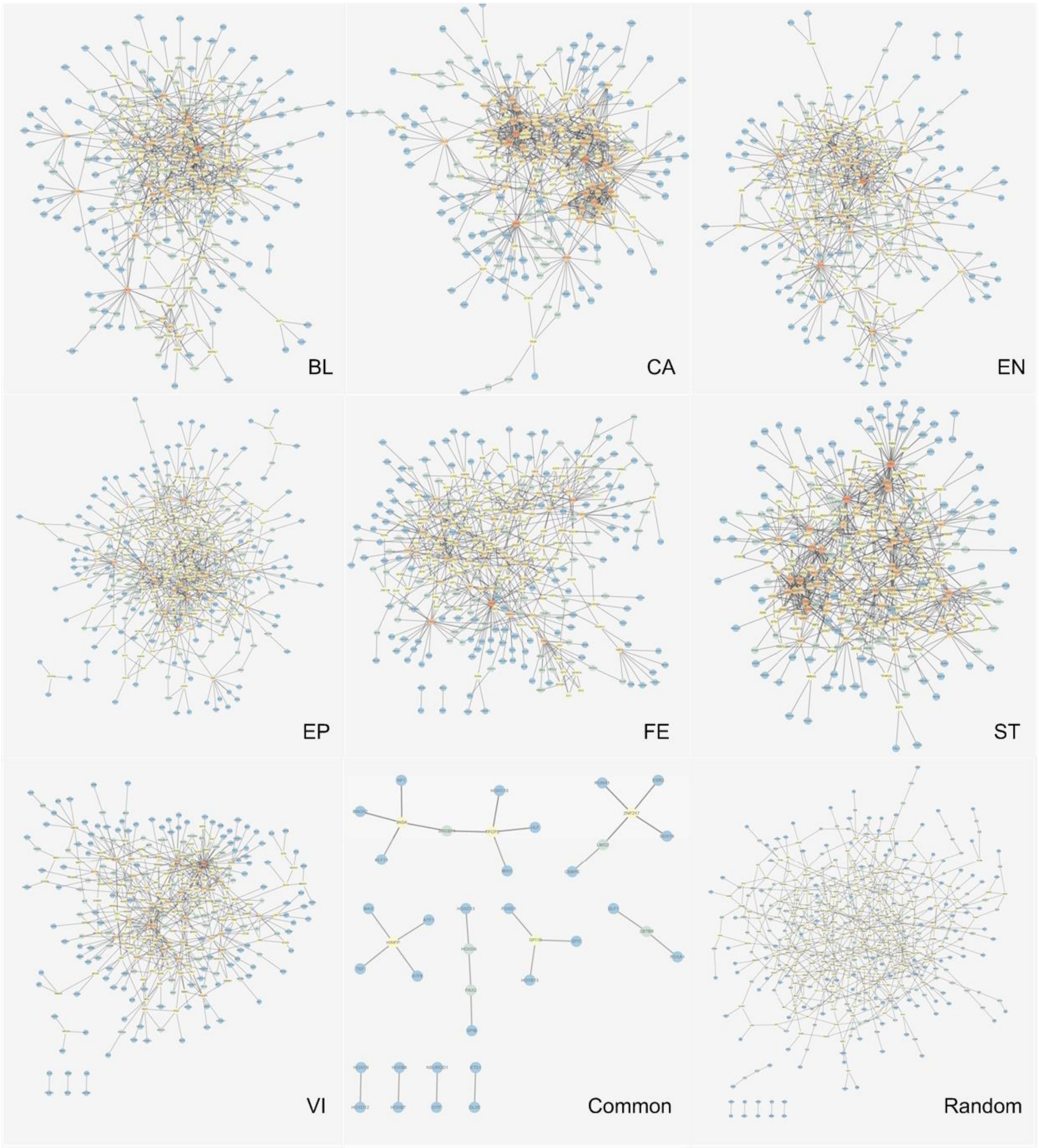

Supplementary Figure 6. Visualization of 7 cell lineage-specific TF interaction networks, common TF interactions, and a random TF interaction network. Abbreviations: random TF interaction network, Random; common TF interactions, Common.

1.2 Legends of Supplementary Tables (see Supplementary tables):

Supplementary Table 1. The list of TFs for the global TF interaction landscape.

Supplementary Table 2. The list of RNA-seq dataset from ENCODE project.

Supplementary Table 3. The list of DNA methylation dataset from ENCODE project.

Supplementary Table 4. The AUC of the integrated networks, three individual ones and integrated ones with any two datasets based on PPI.

Supplementary Table 5. The detail of edges and de novo edge identified from integrative network.

Supplementary Table 6. The function of TF genes in the module (BATF, IRF4, RUNX3, EBF1, PAX5, BCL11A, POU2F2, MEF2A and MEF2C).

Supplementary Table 7. The AUC of 41 cell type interaction networks for every cell lineage.

Supplementary Table 8. AUC of integrative networks (CL-Nets), average-integrative-networks (AI-Nets) as well as the maximum, minimum and average AUC of cell type TF interaction networks (CT-Nets) of the corresponding cell lineage for all seven cell lineages.

Supplementary Table 9. The detail of cell-lineage-specific edges for 7 cell lineages.

Supplementary Table 10. The list of hub TFs identified from cell lineage interaction sets and cell-lineage-specific interactions.
